# Word Spotting as a Service: An Unsupervised and Segmentation-Free Framework for Handwritten Documents

**DOI:** 10.3390/jimaging7120278

**Published:** 2021-12-17

**Authors:** Konstantinos Zagoris, Angelos Amanatiadis, Ioannis Pratikakis

**Affiliations:** 1 Department of Computer Science, Neapolis University, Pafos 8042, Cyprus; k.zagoris@nup.ac.cy; 2 Department of Production and Management Engineering, Democritus University of Thrace, 67132 Xanthi, Greece; 3 Department of Electrical and Computer Engineering, Democritus University of Thrace, 67132 Xanthi, Greece; ipratika@ee.duth.gr

**Keywords:** word spotting, indexing, cloud service, handwritten documents, document-oriented features

## Abstract

Word spotting strategies employed in historical handwritten documents face many challenges due to variation in the writing style and intense degradation. In this paper, a new method that permits efficient and effective word spotting in handwritten documents is presented that relies upon document-oriented local features that take into account information around representative keypoints and a matching process that incorporates a spatial context in a local proximity search without using any training data. The method relies on a document-oriented keypoint and feature extraction, along with a fast feature matching method. This enables the corresponding methodological pipeline to be both effectively and efficiently employed in the cloud so that word spotting can be realised as a service in modern mobile devices. The effectiveness and efficiency of the proposed method in terms of its matching accuracy, along with its fast retrieval time, respectively, are shown after a consistent evaluation of several historical handwritten datasets.

## 1. Introduction

The constant increase in power for mobile-edge computing, along with the availability of various cloud platforms, permits the development of services that were not feasible before for mobile device users [1]. A representative service of this kind is word spotting, which allows for users to retrieve query words of interest in handwritten document images without the need for transcription [2].

The original idea of word spotting was introduced in [3]. It is equivalent to a content-based image retrieval process since it is based on low-level pattern matching, wherein a search in a set of unindexed documents is applied using a query word. In a word spotting context, the image content is the only information source that is used. The outcome of the search is a ranked list of word images that has been retrieved by taking into account the similarity with the query word image. Dealing with document images, word spotting can be viewed as the task of identifying specific locations on a document image that have a high probability to correspond to a queried word image without explicitly recognizing it.

This work is based on an approach that is termed as segmentation-free because the search space is the complete document image, which is contrary to the case that is termed as segmentation-based, wherein the search space is a set of segmented word images. Although a segmentation-free approach is more challenging due to the unconstrained search space that should be dealt with, it has the potential to result in an improved performance in the case of considerable degradation in the document, where the required word segmentation step will introduce many errors, leading to an undesirable erroneous word detection.

For both the aforementioned strategies, the core operational pipeline used relies upon two main components: features extraction and matching. Although there is a plethora of systems that are tailored for both modern [4,5] and historical machine-printed documents [6,7,8,9], in the case of handwritten documents, very few of them can be used effectively due to severe degradation, text layout complexity and variation in the writing style.

The proposed work is an extended version of the work presented in [10,11] using document-oriented local features that consider the information around representative keypoints aiming to address the problem of accessing big data. In this respect, we have introduced a two-folded novelty that comprises a new indexing method, along with a new matching method. Furthermore, we offer a new perspective in the use of word spotting as a service through a mobile device and a cloud service.

**Figure 1:**
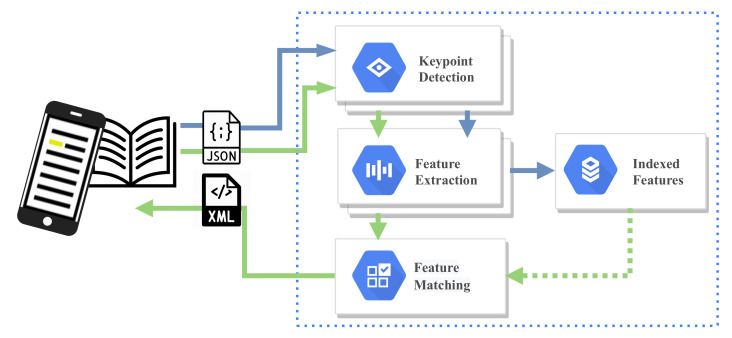
The proposed word spotting client-server pipeline. Blue and green arrows denote the indexing and the query processing sequence, respectively.

Figure 1 shows the communication between a client device and the word spotting. The blue arrow points to the components of the OFFLINE procedure, whereas the green arrow points to the components of the ONLINE procedure, which is described in Section 3. First, the document image is analyzed during the OFFLINE procedure, and the DoLF local keypoints and features are calculated. Then, taking into account DoLF, the corresponding indexed features are created, which are used in the feature matching step during the ONLINE procedure (green arrow) using the corresponding features of the query word image.

## 2. Related Work

Previous approaches to word spotting can be distinguished as supervised or unsupervised based on the need for training data. Furthermore, each approach can be characterized by its dependency on segmentation, resulting in it being considered as either segmentation-based or segmentation-free.

Initial efforts in segmentation-based word spotting [12,13,14,15] used preprocessing techniques, such as binarization and document layout analysis, followed by word image segmentation. Next, for each segmented word image, a set of features is extracted. Finally, taking into account the extracted features, the similarity between the query word image and each segmented word image that has been found in a collection of document images is measured.

Certain works [16,17] on unsupervised word spotting do not require segmentation at either the textline or word level, and are thus denoted as segmentation-free. Instead, they target detecting the most informative parts in the image based on the gradient orientation, and apply a matching procedure based on naive elastic matching. Their main disadvantages are the sensitivity to different writing variations and matching words with different font sizes. Moreover, their matching engine is time-consuming, making its use impractical for large datasets and very costly to provide as a service.

Another similar approach [18] presented a template matching scheme that relies upon a block-based document image descriptor. However, the use of various queries result in introducing much noise in the final merging state from the different queries, especially in the case that variations in the words’ writing and size exist. In the work of Kovalchuk et al. [19], a comparative approach is presented for which they avoid the production of different variations by resizing them to fit a fixed-size rectangle, and, in the sequel, proceed with a HOG and LBP descriptor extraction. Finally, for the retrieval, a nearest-neighbour search is performed, followed by a suppression of extra overlapping candidates.

Apart from the methods mentioned above, which operate in an unsupervised context, many proposed procedures use training data to learn similarities. One such method is Almazan et al. [20], which does not use only word images but also text strings that are both embedded in a common vectorial subspace wherein images and strings representing the same word are found in proximity, which permits treating recognition and retrieval tasks as the nearest neighbour problem. However, the main drawback is the need for many training samples to create a suitable subspace, making it sensitive for unknown words.

Another strategy, aside from using words or the whole document, is by detecting lines. These approaches use Hidden Markov Models (HMMs) models [21] to spot the words inside the lines, embedding a discriminative stage in HMM, such as a support vector machine [22], a neural network [23] or deep learning network architectures [24]. Although they provide more reliable results than the other approaches, they have many drawbacks. Firstly, they required huge amounts of training data, and some cannot identify words that are not present in the training set. Moreover, their dependency on textline segmentation has the same drawbacks as the word segmentation does for degraded documents.

In the work of Llados et al. [25], the performance of various word descriptors in a Bag of Visual Words (BoVW) context is evaluated. They concluded that the statistical approach of the BoVW produces the best performance, although the descriptor storage has significant memory requirements.

Finally, Zagoris et al. [10] presented an unsupervised word detection method that employed novel local features, namely, Document-oriented Local Features (DoLF), whose texture information is considered in a wider neighborhood spatial context. Both segmentation-based and segmentation-free scenarios have been considered. Although they provide superior results for an unsupervised approach, their matching procedure is not efficient for large document sets, and thus they are prohibited to being provided as a cloud service.

This paper proposes a holistic unsupervised segmentation-free method for word spotting, addressing the limitations of the works mentioned above that are suitable for cloud service. It employs the DoLF local features from [10] on preprocessing documents using a completely different and dynamic matching procedure suitable for efficient results on big datasets without compromising the effectiveness.

## 3. Proposed Methodology

**Figure 2:**
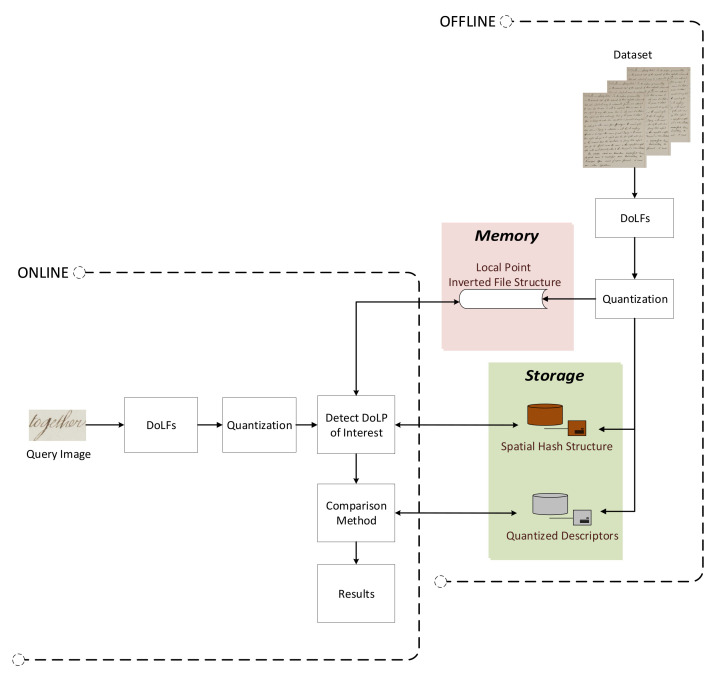
The proposed word spotting architecture. The OFFLINE procedure is only performed once to create the appropriate data structures. The ONLINE process is visible to the user and executes when locating the word.

Figure 2 shows the proposed methodology. First, the DoLF local features are calculated and detected on a preprocessing document image. Afterwards, an indexing procedure uses the calculated DoLF and creates a set of data structures that allow for efficient and effective word spotting. Next, the user selects the query word image on which the DoLF local features are calculated. Finally, a novel matching procedure called Quantitative Near Neighborhood Search (QNNS) detects visually similar regions to this query word image on documents and presents them to the user.

### 3.1. Preprocessing and Local Points Calculation

The initial step contains a pre-processing step that aims to enhance the contrast between the foreground and background pixels of the document image and to keep the valuable edge boundary information. This enhances the effectiveness of the subsequent calculation of the gradient-based keypoints and descriptors. The required pre-processing step is the only operation that runs on-board on the client application, and comprises two steps. The first step consists of a contrast normalization method [26], which deals with the illumination changes imposed by the mobile device and the user. Subsequently, the gradient vectors $I_{x}$ and $I_{y}$ of the document image *I* are calculated and filtered by a high pass filter for eliminating any remaining background noise. The filter thresholds are calculated dynamically based on the Otsu algorithm for minimizing the intra-class variance between the foreground and background pixel clusters.

**Figure 3:**
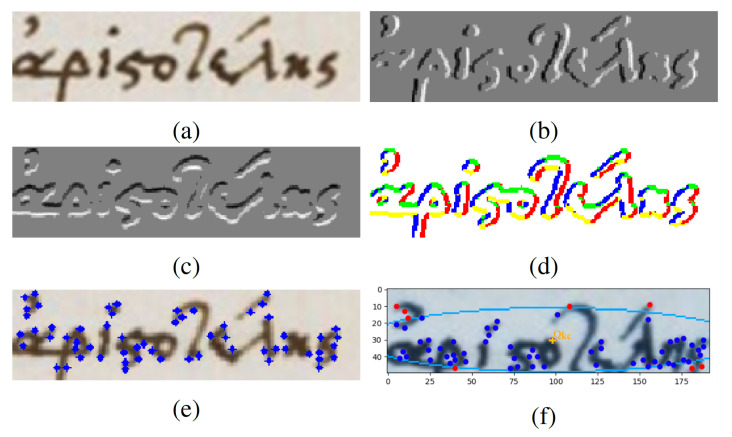
The proposed word spotting indexing and matching pipeline for the Greek handwritten word of ’Aristotle’. (**a**) Query image; (**b**) filtered gradient image $I_{x}$; (**c**) filtered gradient image $I_{y}$; (**d**) quantization of the gradient orientation; (**e**) keypoints; (**f**) query keypoint (yellow) and the ellipse area enclosed keypoints (blue) that should be matched during the matching process.

The two filtered gradient images, as shown in Figure 3a,b, are then encoded in a JavaScript Object Notation (JSON) format and passed to the server application for the keypoint extraction.

For the keypoint and feature extraction, we adapted a version of the Document-oriented Local Features (DoLF) proposed by the authors [10]. While most works that use local features are based on the Scale Invariant Feature Transform (SIFT) [27], which is initially used for natural images, in comparison with the document images, they have many structural differences that create problems, such as:

- The erroneous local points detection between the document lines due to the image pyramid scaling;
- The invariant properties of those descriptors that amplify noise. As stated in [17], features that are invariant to rotation result in a worse performance compared to features that depend on rotation. Furthermore, they adhere to the observation that invariant-to-rotation features are more sensitive to the noise.

**Figure 4:**
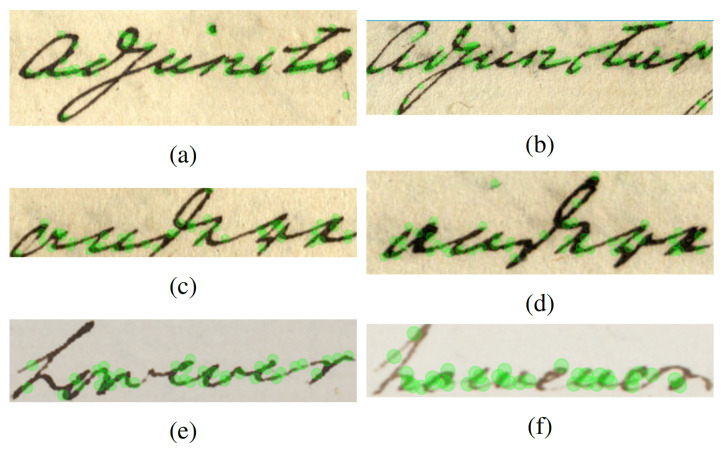
Presentation of the Dolf’s location for various handwritten words: (**a**) ‘adjuncto’; (**b**) ‘adjunctur’; (**c**,**d**) ‘andere’; (**e**,**f**) ‘however’.

On the contrary, the DoLF exhibit some desirable characteristics that make them suitable for the proposed method, such as:

- They take into consideration the handwritten document particularities;
- They provide consistency between different handwritten writing variations, as shown in Figure 4;
- Their descriptors contain texture information in a spatial context, which is suitable in dealing with a document collection created by different writers, containing significant writing style variations.

The DoLF computation comprises two parts: the keypoint detection and the feature calculation around it. Subsequently, a brief description of the keypoints and their descriptors is provided. For a detailed description, the work in [10,28] should be consulted.

The next step involves the linear quantization of the gradient orientation. This step aims to label the changes to the writing direction, as these points consist of important and descriptive information. Figure 3d shows the output for the quantization of the gradient orientation values. Each color represents a different quantization level. The current work uses the default four different quantization levels as presented in [10,28].

Next, for each quantization level, the Connected Components (CC) are detected. These CCs represent chunks of strokes that correspond to different writing directions. The final local points are the center of gravity of each remaining CC. An example of these keypoints is shown in Figure 3e. The keypoint calculation method can detect meaningful points of the characters that reside in the documents independently of its scale. Moreover, it provides some consistency between different handwritten writing variations.

**Figure 5:**
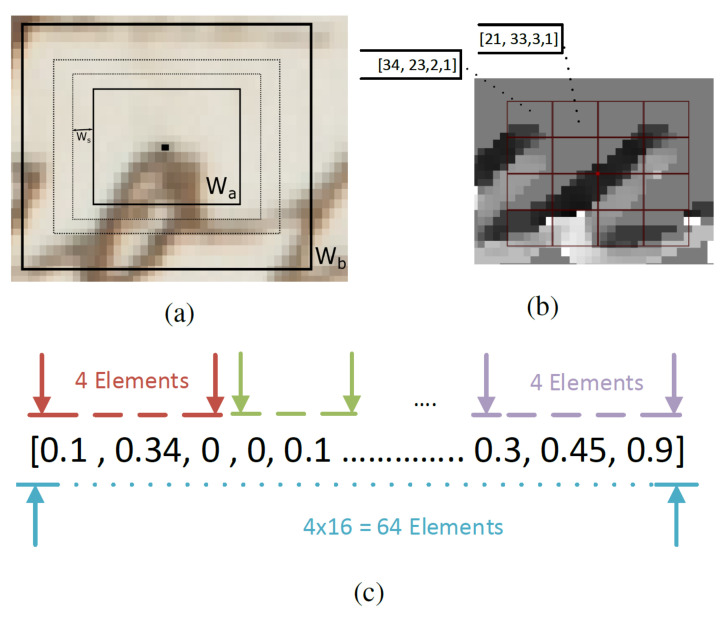
Feature extraction: (**a**) scale invariant window size definition; (**b**) features calculation; (**c**) descriptor structure.

The next step involves the calculation of the features around the detected keypoints. The descriptor is calculated upon a scale-invariance window size around the detected local points, as shown in Figure 5a. The window size is defined dynamically by calculating the mean brightness in different window sizes and selecting the one that has the maximum value, as described in [29,30].

Then, the selected window size is divided into 16 cells, where a four-bin histogram is calculated, representing each cell (each bin corresponds to a quantization level). Each pixel inside a cell accumulates a vote in the corresponding angle histogram bin, as shown in Figure 5b. The strength of voting depends on the pixel’s magnitude of the gradient vector.

All histograms are concatenated in a single 64-bin histogram and normalized by its norm. Finally, all values above 0.2 are set to 0.2 and are re-normalized again to minimize the illumination effect in the descriptor [27]. The final descriptor is shown in Figure 5c.

### 3.2. Features Indexing Method

The sheer magnitude of the detected local points and their descriptors increases the costs in terms of time, memory and storage requirements, rendering them unusable for providing a service. To solve this issue, we transform the information from the DoLF to several different structures that permit efficient word spotting without compromising on the effectiveness.

The proposed indexing method comprises two distinct steps:

- A descriptor quantization using multiple Bag of Visual Words (BoVW) to decompose and compress its information;
- Three different memory and storage structures for very quickly segmentation-free word spotting during the client request.

**Figure 6:**
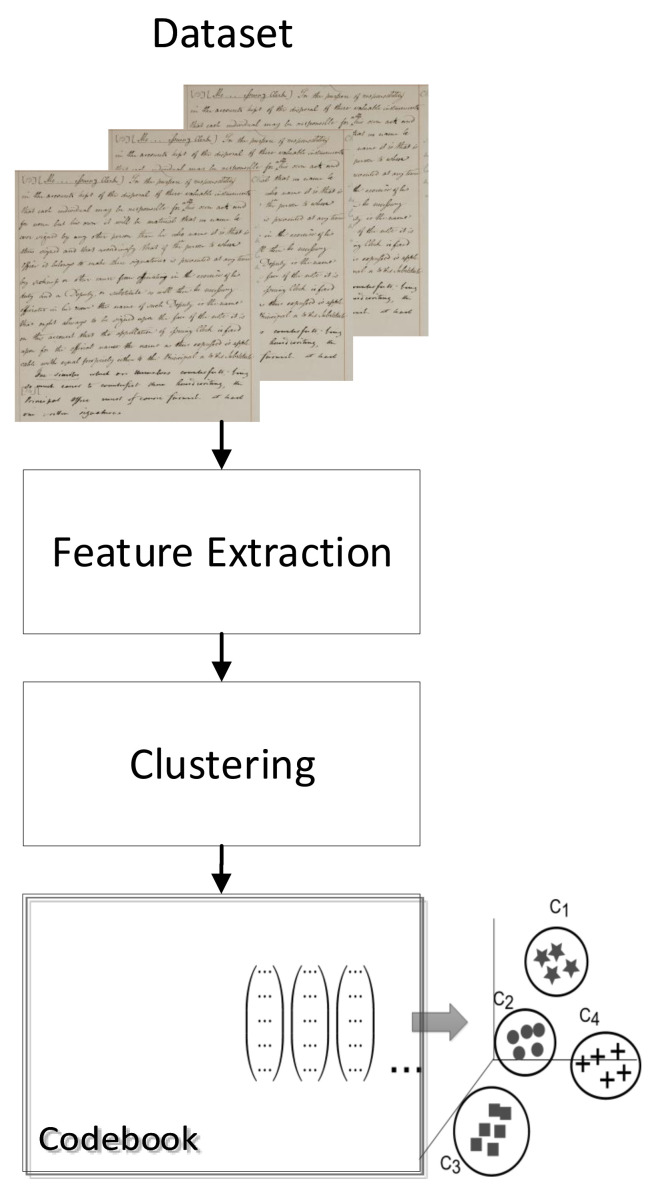
The overall architecture for the quantization of the DoLF descriptors using multiple Bag of Visual Words.

Figure 6 shows the overall architecture of the quantization based on Bag of Visual Words.

#### 3.2.1. Descriptor Quantization

At this point, the descriptor is split into four different parts. This is because each part describes a different spatial part of the descriptors. Next, four different codebooks are created for the different parts of the descriptor.

The size of each codebook corresponds to the descriptor precision, but with increasing processing and storage costs. For our experiments, a codebook of size 16 is used. The final quantized descriptor is a four-bin histogram, with each bin in the range of 0–15 values.

#### 3.2.2. Indexing Data Structures

**Figure 7:**
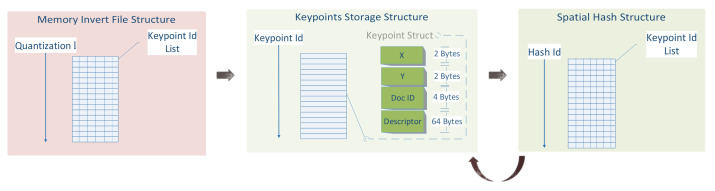
Memory and storage structures and their relationships.

Then, three data structures are created to facilitate the low retrieval times, incorporating the information from the local points location and the corresponding quantized descriptor. The memory invert file structure, the descriptors storage structure and the spatial hash structure are shown in Figure 7.

The Keypoints Storage Structure (KSS) is the simplest and most prominent data structure. It contains all of the calculated keypoints and their descriptors. It is a struct array in which the array index corresponds to the keypoints Id. Each struct (72 bytes) holds the keypoint location (*X*, *Y*), the document that resides (DocID) and its quantized descriptor.

**Figure 8:**
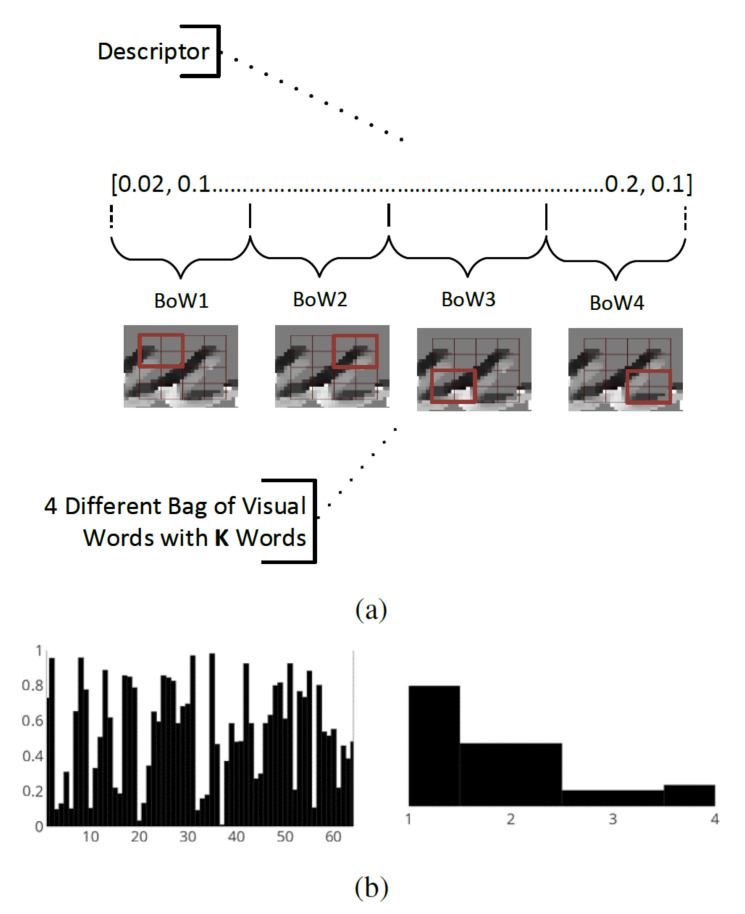
(**a**) The visual representation for the descriptor quantization using four different Bag of Visual Words; (**b**) the descriptor histogram, before and after applying quantization.

The Memory Invert File Structure (MIFS) resides in memory. It is an array of 64-bit integer lists (8 bytes). Each descriptor corresponds to a hash id *l*, which is calculated from the following hash function:

(1) $$l=BOW_{1}*K^{3}+BOW_{2}*K^{2}+BOW_{3}*K+BOW_{4}$$

where $BOW_{i}$ corresponds to each quantized value of the descriptors (Figure 8a) and *K* is equal to the codebook size. In our proposed method, K=16.

The MIFS is a dictionary-based structure that maps a list of keypoints *K* with the same hash value *l*. Thus, the MIFS primary function is to retrieve a list of descriptors *D* that have the same hash value *l*.

The Spatial Hash Structure (SHS) assists the detection of every keypoint that resides in a specific location inside a document, as it provides information about the spatial distribution of the local points inside a document. Its primary function is to retrieve a list of descriptors *D* whose local points location is close to each other inside the document.

This is achieved by using the following hash function:

(2) $$h\left(x,y,n\right)=n*A^{2}+y*A+x$$

where *A* is any custom-defined number that is greater than the maximum width and height document inside a collection.

The above hash function takes the location of the keypoint (*x*, *y*) and the document *n* as an input, resulting in the hashID computation of the corresponding local point.

The retrieval of all of the DoLF in proximity to the point (x1,y1) is achieved by:

First, calculating the hashIds for all of the local points residing inside the space (x±dx,y±dy) for the document *n*. The dx and dy denote the distance space from the local point (x1,y1). Next, all of the descriptor IDs are retrieved from the SHS that corresponds to that specific hashIds. Finally, through the KSS, all of the descriptor information is available.

These three storage structs are the minimum required information to be stored from a documents’ collection. Only the MIFS needs to reside in memory; the other two can be stored in disks and accessed when needed.

### 3.3. Feature Matching

Feature matching is the only procedure that affects the user, as it commences during the word spotting search. Initially, the query image is analyzed, and the DoLF is calculated. From them, the quantized descriptors and the corresponding hash values *l* are calculated based on the previous trained BoVW. Finally, the hash values *l* are calculated based on the Equation (1) for each DoLF.

Through the MIFS, all of the descriptor IDs with the same hash value *l* with the query *l* value are retrieved. Finally, their locations and descriptors (denoted as $descriptors_{n}$) are retrieved through KSS.

The feature matching goal is to identify those keypoints with a similar spatial distribution and descriptors with the query keypoints.

First, the nearest keypoint $Qk_{c}$ from the $\left(c_{x},c_{y}\right)$ point is identified. The $\left(c_{x},c_{y}\right)$ is denoted as the mean center $\left(c_{x},c_{y}\right)$ of the keypoints set in the query word image, and is calculated as:

(3) $$\left(c_{x},c_{y}\right)=\left(\frac{\sum_{i=1}^{k}p_{x}^{i}}{k},\frac{\sum_{i=1}^{k}p_{y}^{i}}{k}\right)$$

where $p_{x}^{i},p_{y}^{i}$ denote the location of the *i*th keypoint and *k* denotes the total number of the keypoints in a word image.

The next step involves identifying the most similar local points with the $QK_{c}$ from the retrieved $descriptors_{n}$.

In our implementation, the Euclidean Distance (ED) is used, and the top *N* matches that are kept denote those that have a distance from the query keypoint $Qk_{c}$ feature equal to those that have a distance from the query keypoint $Qk_{c}$ feature lower than a threshold *t*. This threshold is experimentally defined and controls the time expense of the search in the document space. Each keypoint that belongs to the top N matches is a document candidate coordinate origin similar to the query image.

The spatial NNS for each keypoint that resides on the query image is addressed in the next stage. The spatial NNS is realized in a search area around each point. During the search, if there are one or more keypoints in the proximity of the query keypoint under consideration, the Euclidean distance between their descriptors is calculated and the minimum distance is kept. The previous procedure is repeated for each keypoint in the query image. The final similarity measure is the average of all of the minimum distances. In the case that a local point in its proximity does not exist, then the query local point is ignored.

As a final stage, the system presents all of the word images based on an ascending sort order of the calculated similarity measure. The server-side returns the user’s results in ascending order in an Extensible Markup Language (XML) format.

## 4. Experimental Results

The proposed method and corresponding service were evaluated on the following four different handwritten document collections:

1. *English* dataset that contains 115 pages/images and 15,923 words;
2. *German* dataset that contains 100 pages/images and 15,579 words;
3. *Finnish* dataset that contains 56 pages/images and 16,201 words;
4. *Greek* dataset that contains 120 pages/images and 18,809 words.

**Figure 9:**
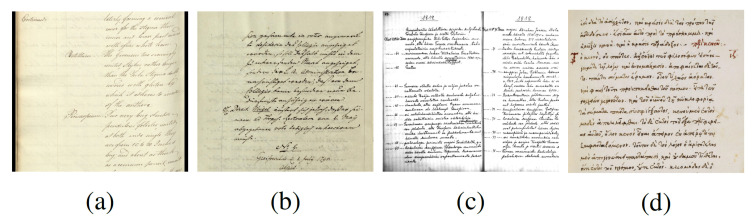
Representative document images from (**a**) English dataset; (**b**) German dataset; (**c**) Finnish dataset; (**d**) Greek dataset.

Figure 9 shows a representative document image from each dataset.

Initially, the proposed method is evaluated against the original method [10] that used the DoLF local points, which was superior to most unsupervised word spotting approaches. Table 1 shows that the proposed method and the original achieve a similar performance, while the novel indexing scheme used in the proposed method results in a compression of the available information from DoLF, thus enabling an efficient and effective word spotting on big datasets.

In the sequel, our experimental work contains a comparison between the proposed method and the original method in terms of time, memory and storage requirements, which are essential for reducing the running cost for the word spotting service. Since the original method has extensive storage and memory requirements when used in a production environment, it becomes unsuitable for large datasets. Table 2 shows that the proposed method provides a massive reduction in the retrieval time per query, memory requirement per document and storage requirement without compromising its performance, as shown in Table 1.

Finally, aiming to demonstrate how the proposed method will scale up based on the dataset size, we record the time, memory and storage requirements with different dataset sizes, namely, 50, 5000 and 50,000 documents, respectively. Table 3 shows that the retrieval time per query increases in a non-linear manner, making the search feasible in terms of time consumption for large scale datasets.

Table 4 shows the results for the ICDAR 2015 Competition segmentation-free TRACK I.B., which involves 1421 queries. It is worth noting that the marginally better performance of the PRG group uses a word segmentation algorithm, creating a dependency from the context of a segmentation-based scenario. Therefore, with a more complicated document layout, the PRG group’s performance will decrease considerably. However, in our proposed segmentation-free method, there is no performance loss despite extensive compression due to the proposed indexing.

## 5. Conclusions

In this paper, we present an effective and efficient word spotting method for handwritten documents that permits supporting a cloud-based service-oriented perspective in the use of word spotting. After employing a consistent experimental work, we have shown that the proposed method considerably decreases the retrieval time and the overall memory and storage requirements without a substantial performance loss. The improved efficiency, along with the appealing effectiveness achieved, enables word spotting to be addressed as a mobile device service that relies on the lack of any training dependency and its novel fast and spatial feature matching process.

## Figures and Tables

**Table 1:** Experimental results for four handwritten segmentation-free datasets.

Method	English		German		Finnish		Greek	
	P@5	MAP	P@5	MAP	P@5	MAP	P@5	MAP
Original [10]	0.35	0.22	0.59	0.42	0.58	0.43	0.39	0.31
Current	0.35	0.22	0.57	0.38	0.56	0.39	0.40	0.32

**Table 2:** Time, memory and storage requirements.

Method	Retrieval Time per Query (sec)	Memory Requirement per Document (KB)	Storage Requirement per Document (KB)
Original [10]	15.84	19800	19800
Current	0.66	49	1676

**Table 3:** Comparative evaluation results for different size datasets.

Database (Documents)	Retrieval Time per Query (sec)	Overall Memory Requirement (MB)	Overall Storage Requirement (MB)
50	0.61	2.1	69
5000	0.89	213	2693
50,000	1.1	448	5843

**Table 4:** Experimental results for Track I.B.—training and segmentation-free (ICDAR15 competition).

Method	P@5	MAP
PRG Group	0.376	0.293
CVC Group [31]	0.150	0.116
Competition Baseline [5]	0.150	0.116
Original [10]	0.387	0.326
Proposed Method	0.3521	0.282
